# Efficacy of an app-based treatment for anxiety disorders including exposure in virtual reality – a randomized controlled trial

**DOI:** 10.1192/j.eurpsy.2023.300

**Published:** 2023-07-19

**Authors:** A. K. Rubart, T. F. Barrabas, I. K. Manheim, J. Korn, J. Berg, L. H. John, B. Zurowski

**Affiliations:** 1 Department of Psychiatry and Psychotherapy, University of Lübeck, Lübeck; 2 Department of Psychiatry and Psychotherapy, Kiel University, Kiel, Germany

## Abstract

**Introduction:**

Anxiety disorders are among the most prevalent mental disorders. However, only a minority of patients receives adequate psychotherapeutic treatment despite strong empirical evidence for the efficacy of CBT in anxiety disorders (Marcks *et al.* Psychiatr Serv 2009; 60 823-830). App-based psychotherapy can help to reduce this massive treatment gap.

**Objectives:**

We aimed at evaluating the efficacy of an app-based treatment for anxiety disorders including exposure in virtual reality.

**Methods:**

The randomized controlled trial was conducted in two university outpatient treatment centers in Northern Germany. Patients were diagnosed with agoraphobia (AP; with or without panic disorder; n=103), panic disorder (PD; n=84) or social anxiety disorder (SAD; n=110) and were randomly assigned to either the app-based intervention or treatment as usual (up to 6 sessions of supportive therapy). The app was developed based on evaluated CBT manuals and includes 14 hours of audio and video content and 15 disorder specific virtual reality exposure scenarios. Participants in the intervention groups also received two appointments with a therapist during the app-based treatment. Primary outcome was the change in Beck Anxiety Inventory (BAI) score pre to post (after 6 months). Mixed ANOVAs were conducted in intention to treat and completer analyses. Secondary outcomes were disorder specific questionnaires (Liebowitz Social Anxiety Scale LSAS for SAD and Panic and Agoraphobia Scale PAS for AP and PD) and health related quality of life measured with a single item (L-1).

**Results:**

In the ITT analysis, the interaction effect between group and time was significant in patients with AP as well as in patients with PD (AP: p=.014, partial η²=.06; PD: p=.028, partial η²=.06). This indicates a stronger improvement of symptoms in the intervention group compared to the control group. In patients with SAD, there was no significant interaction effect (p=.101, partial η²=.03). The disorder specific measures LSAS and PAS showed a significantly stronger decrease in the intervention group than in the control group for each of the specific disorders. Concerning quality of life, a stronger improvement in the intervention group was only found in patients with PD.

**Conclusions:**

A stronger symptom reduction in the app-based intervention group compared to the control group could be found in patients with AP (BAI/PAS), PD (BAI/PAS) and SAD (LSAS). This is particularly remarkable as the app was compared to an active control group with up to 6 sessions of psychotherapy. Effect sizes were comparable to those found in studies comparing face-to-face CBT to an active control group. The lack of an intervention-specific effect on BAI scores in patients with SAD might be due to the poor sensitivity of the BAI for the specific symptoms of SAD.

**Disclosure of Interest:**

None Declared

